# Optimization and use of near infrared imaging to guide lymph node collection in rhesus macaques (*Macaca mulatta*)

**DOI:** 10.1111/jmp.12605

**Published:** 2022-07-15

**Authors:** Jeremy V. Smedley, Rachele M. Bochart, Miranda Fischer, Heidi Funderburgh, Vanessa Kelly, Hugh Crank, Kim Armantrout, Oriene Shiel, Mitchell Robertson‐LeVay, Nikki Sternberger, Brian Schmaling, Sheila Roberts, Vicki Sekiguchi, Michael Reusz, Tiah Schwartz, Kimberly A. Meyer, Gabriela Webb, Roxanne M. Gilbride, Nicholas Dambrauskas, Daniela Andrade, Matthew Wood, Caralyn Labriola, Michael Axthelm, Nina Derby, Ben Varco‐Merth, Yoshinori Fukazawa, Scott Hansen, Jonah B. Sacha, Donald L. Sodora, D. Noah Sather

**Affiliations:** ^1^ Infectious Disease Resource, Division of Pathobiology and Immunology Oregon National Primate Research Center, Oregon Health and Science University Beaverton Oregon USA; ^2^ Surgical Services Unit Oregon National Primate Research Center, Oregon Health and Science University Beaverton Oregon USA; ^3^ Center for Global Infectious Disease Research Seattle Children's Research Institute Seattle Washington USA; ^4^ Vaccine and Gene Therapy Institute, Oregon Health and Science University Beaverton Oregon USA; ^5^ Experimental Pathology Unit, Division of Pathobiology and Immunology Oregon National Primate Research Center, Oregon Health and Science University Beaverton Oregon USA; ^6^ Department of Pediatrics University of Washington Seattle Washington USA

**Keywords:** draining, fluorescence, ICG, imaging, indocyanine green, lymph node, macaque, mesenteric, MLN, targeted sampling

## Abstract

**Background:**

Identification of lymph nodes (LNs) draining a specific site or in obese macaques can be challenging.

**Methods:**

Indocyanine Green (ICG) was administered intradermal (ID), intramuscular, in the oral mucosa, or subserosal in the colon followed by Near Infrared (NIR) imaging.

**Results:**

After optimization to maximize LN identification, intradermal ICG was successful in identifying 50–100% of the axillary/inguinal LN at a site. Using NIR, collection of peripheral and mesenteric LNs in obese macaques was 100% successful after traditional methods failed. Additionally, guided collection of LNs draining the site of intraepithelial or intramuscular immunization demonstrated significantly increased numbers of T follicular helper (Tfh) cells in germinal centers of draining compared to nondraining LNs.

**Conclusion:**

These imaging techniques optimize our ability to evaluate immune changes within LNs over time, even in obese macaques. This approach allows for targeted serial biopsies that permit confidence that draining LNs are being harvested throughout the study.

## 1 INTRODUCTION

One of the key advantages of macaque models is the ability to perform in‐depth tissue sampling in ways that are not possible in human patients. To this end, we had previously developed minimally invasive (MIS) laparoscopic techniques to permit sampling of key immunologic sites,^1^, ^2^, ^3^, ^4^ which permit further characterization of tissue responses to infectious diseases and immunology in the macaque model. One important site that we have sampled in the past is mesenteric lymph nodes (MLNs) which are important reservoir sites for SIV and are immunologically distinct from peripheral lymph nodes (PLN)^3^, ^5^, ^6^, ^7^, ^8^, ^9^, ^10^ due to their exposure to microbes and microbial products coming from the GI tract. These sites had previously been difficult to sample serially, but our MIS techniques have been used to sample them at up to eight timepoints during the course of a study, with the main obstacle to successful collection being the difficulty to identify MLN in obese animals.

Sampling of peripheral lymph nodes in macaque models can also present challenges, with the need to sample sites repeatedly combined with factors such as obesity; LNs can be hard to find resulting in missed time points. As macaque studies are typically only powered sufficiently to detect differences when all samples are evaluated, missed time points can impact the ability to achieve meaningful and statistically significant results. Additionally, for events that are localized in nature, such as immunization at a specific site, it can be important to ensure that the LNs collected are actually draining the site of interest. Drainage patterns can vary by individual^11^, ^12^ and nondraining LN (LNs not draining the site of interest) from the same PLN site (axillary, inguinal, submandibular, etc.) can easily be inadvertently collected unless targeted collection is performed.

To address issues associated with accuracy of lymph node collections, we have optimized a dye‐guided lymph node (DGLN) collection technique for use in the macaque model. This technique utilizes the dye indocyanine green (ICG) which is visualized with near infrared (NIR) imaging to allow for 1. Targeted collection of LNs from specific sites, and 2. Identification of LNs in obese animals where they were not identifiably using standard methods. Here, we present the DGLN technique for identification of MLN in obese animals and the identification of LNs draining the site of immunization in both the oral mucosa (intraepithelial or IEp inoculation) and bicep muscle (IM inoculation). Draining LNs were compared to nondraining LNs from these sites (axillary LNs for IM and submandibular LNs for oral mucosa IEp) following immunization and significant differences in replication and levels of key immune cells within germinal centers were identified.

## 2 MATERIALS AND METHODS

### 2.1 Humane care guidelines

All animals were housed, cared for, and used in accordance with the policies of the Institutional Animal Care and Use Committee (IACUC) at the ONPRC, an AAALAC‐accredited institution, which abides by the USDA Animal Welfare Regulations, the Public Health Service Policy on Humane Care and Use of Laboratory Animals and the Guide for the Care and Use of Laboratory Animals. All enrolled macaques were SPF (serologically negative for SIV, simian type D retrovirus, and Macacine herpes 1) prior to the start of the study and were captive‐born at ONPRC. All animals were singly or socially housed in accordance with their IACUC protocol. Throughout the study, all animals were uniformly fed Purina LabDiet 5000 (Purina Mills International), daily nutritional enrichment items (grains, fruits, or vegetables) and had ad libitum access to water. In addition to social housing and nutritional enrichment, animals received environmental enrichment in the form of toys, music/radio, and television daily. All animals were observed for health or behavioral concerns at minimum twice daily by research, veterinary, and/or husbandry staff. Animals requiring euthanasia for study or clinical endpoint purposes were sedated with an intramuscular injection of 20 mg/kg ketamine HCl (Ketathesia™, Henry Schein Animal Health) followed by an overdose of intravenous pentobarbitol solution, in accordance with the AVMA Guidelines for the Euthanasia of Animals.

Nineteen rhesus macaques (15 males/4 females), ranging from 3 to 16 years of age and 5.6–12.1 kg were used to optimize ICG administration for fluobeam PLN detection. Dose volume, number of injection sites, and duration post‐administration were analyzed to optimize LN signal identification for inguinal and axillary LN sites. Collection of MLN and PLN using ICG guidance in obese rhesus macaques was performed as part of their primary study once standard methods had failed to identify an MLN and/or PLN at that timepoint. Thirteen obese rhesus macaques (7 males/6 females) ranging from 7 to 17 years old and 7.5–16.1 kg were evaluated; 7/13 were used for PLN, and 9/13 were used for MLN detection. A total of 2 rhesus (females, 10–11 years old, and 7–8.5 kg) macaques (n = 1/group) were injected with ICG labeled 10 014_F8.V3.N7.SOSIP (natively HIV envelope folded trimer) either by IM injection in the biceps brachii or IEp injection in the oral mucosa. A boost injection at the same site was performed 4 weeks after the initial immunization, and the draining LNs (submandibular for the oral site, axillary for the IM) were collected 2 weeks after the boost. Unbound ICG was also injected at the site of immunization prior to surgery to ensure that sufficient signal was present in the draining LNs for identification by NIR. Nondraining (no fluorescent signal) LNs from the same site (axillary for biceps brachii IM and submandibular for oral mucosal vaccine administration) were collected at the same time for comparison to the draining LNs.

### 2.2 Administration of ICG and NIR imaging

ICG (Diagnostic Green_LLC_) was reconstituted with sterile water to 5 mg/mL and then diluted with sterile saline to 0.625 mg/ml concentration prior to use. Macaques were sedated with an intramuscular injection of 10–12 mg/kg ketamine HCl (Ketathesia™, Henry Schein Animal Health) and 0.015 mg/kg dexmedetomidine hydrochloride (Dexmedesed™, Dechra). ICG was administered through a 25‐27G needle at volumes of 0.1–0.4 ml per site intradermal (ID), IEp in the oral mucosa, IM in the biceps brachii, or subserosal in the colon to identify draining LNs using NIR imaging. Real‐time NIR imaging was performed using a Fluobeam800 for PLN and a Stryker 1688 for laparoscopic procedures.

For the inguinal LN site, the lower caudal abdomen and proximal medial thigh were sites of ICG administration; for the axillary LN site, the proximal medial brachium and anterolateral aspect of chest were sites of ICG administration. ICG injection sites were selected approximately 5 cm from the expected location of axillary/inguinal LNs to avoid overlap of the NIR signal from the injection site with the expected location of the LNs. The Fluobeam800 was positioned 20 cm from the patient, and 0.1–0.2 ml of ICG (0.625 mg/ml) ID was administered at 1 or 2 sites. The Fluoptics Fluorescence Imaging for Surgery (Fluobeam Software v. 4.0) was utilized (Zoom: 1, Brightness: 25, Exposure: 40, Background 5–6, Focus Auto, and White light: off) to capture 5, 10, and 15 minute time points. Image fluorescence were quantified by pixel threshold analysis using ImageJ software (1.53 m September 28, 2021, rsb.info.nih.gov/ij) to determine the pixel area of fluorescent signal to determine optimization for the technique.

A Stryker 1688 AIM 4 K Platform with a Stryker L11 LED light source and a Stryker AIM HD Laparoscope, 5.4 mm, 0°, 30 cm was used for laparoscopy. The SPY gain settings, which determine the strength of the fluorescence signal, ranged from 1 to 8, and brightness at 1–3 for fluorescence imaging.

### 2.3 Administration of SOSIP trimer

10 014_F8.V3.N7.SOSIP Envelope protein was expressed in HEK293 cells and purified by GNA lecting chromatography as previously described.^13^ The sequence is derived from an *env* sequence isolated from an HIV‐1+ subject (10014) that developed broadly neutralizing antibodies approximately 3 years post‐infection (cite 25 122 781). For conjugation, protein was mixed with NH‐Reactive ICG and Reaction Buffer and incubated for 10 min at 37°C. The reaction was stopped with WS buffer and filtered, and the labeled protein was diluted into phosphate‐buffered saline (PBS). Labeling efficiency was determined by assessing absorbance at 280 nm and 800 nm and calculating the absorption coefficient. ICG‐labeled Env vaccine formulation was prepared with the following adjuvants so that each injection contained 100ug Env trimer, 100ug R848 (Invivogen), 10ug MPLA (Invivogen), and 1% Alhydrogel (Invivogen). The labeled SOSIP trimer was injected in a volume of 0.1 ml/site into the oral mucosa of both the left and right cheek pouch. For IM, a volume of 0.25 ml was injected into the right and left biceps brachii.

### 2.4 Biopsy procedure

Biopsies were performed as described previously.^3^, ^4^ Briefly, animals were fasted overnight and sedated with an intramuscular injection of 10–12 mg/kg ketamine HCl (Ketathesia™, Henry Schein Animal Health) and 0.015 mg/kg dexmedetomidine hydrochloride (Dexmedesed™, Dechra). Some animals were also intubated and placed on isoflurane (0.8%–2%) if they received mucosal or laparoscopic biopsy procedures. Once anesthetized, all animals received an IM injection of buprenorphine HCl (Buprenex®, Reckitt Benckiser; 0.3 mg/animal weighing >3 kg; 0.15 mg/animal 1.5–3 kg; 0.04 mg/kg/animal <1.5 kg) or a subcutaneous (SC) injection of sustained release buprenorphine (Buprenorphine SR, ZooPharm; 0.2 mg/kg/animal). Once prepared for surgery, sites were draped and all procedures were performed using aseptic technique. Incisions were made with a #15 surgical blade and biopsy sites were closed in 2–3 layers depending on location, using 4–0 absorbable monofilament suture. Axillary and inguinal sites were closed in two layers (subcutaneous and skin), while submandibular and laparoscopic port sites were closed in three layers (muscle, subcutaneous, and skin). All skin closure was performed using an intradermal pattern, followed by application of cyanoacrylate tissue adhesive (Vetbond™ Tissue Adhesive, 3 M). Instillation of local anesthesia along the incision was also performed.

### 2.5 Cell counts

PLN and MLN were obtained as described above and were processed then stained for flow cytometric analysis. Lymph nodes were collected in R10 media (RPMI‐1640 with 10% sterile filtered Newborn Calf Serum, 1% penicillin–streptomycin, 1% L‐glutamine, 1% sodium pyruvate, and 50 nM/mL of 2‐Mercaptoethanol) at biopsy and were then processed using a pestle and screen for CD‐1 (Sigma‐Aldrich, S1020‐5EA) in a tissue culture dish. Processed tissues were strained through a 40 μm filter into a 50 ml conical, centrifuged at 700 rcf for 10 minutes, and resuspended in R10 before obtaining the cell yield using a Horiba ABX Pentra 60 C+ hematology analyzer.

### 2.6 Immunofluorescence microscopy of lymph nodes

Formalin‐fixed, paraffin embedded 5 μm sections were deparaffinized and decloaked in 1% citrate buffer at pH 6.0 (Vector Laboratories). Sections were blocked in 1%BSA with goat serum prior to staining for CD3 (I nvitrogen, SP7, 1:150), PD‐1 (Abcam, NAT105, 1:200), and CD20 (Abcam, EP459Y, 1:300) overnight. Following primary staining, tissues were washed and incubated with goat anti‐mouse Alexa Fluor 488 (Invitrogen, 1:500) and goat anti‐rabbit Alexa Fluor 594 (Invitrogen, 1:500) for 1 hour at room temperature. Background signal was quenched by 1‐minute incubation using Vector TRUEView Autofluorescence Quenching Kit (Vector Laboratories). Coverslips were mounted, and nuclei stained using Vectashield Hardset Antifade Mounting Medium with DAPI (Vector Laboratories). Composite tissue images were acquired on a Keyence BZ‐X710 fluorescence microscope at 20× magnification. Identification of active germinal centers and T‐cell quantification were performed using open‐source packages available in FiJI.

### 2.7 Statistical analysis

All data are shown as standard deviation (SD) of the mean. Optimization results were analyzed using paired t‐tests for comparison of signal intensity at different times within an animal and unpaired t‐tests for comparison of volume and number of sites using GraphPad Prism (San Diego, CA, USA) version 9.0 for Windows. Results were considered statistically significant when the *p* value was less than .05.

## 3 RESULTS

### 3.1 Optimization of ICG for axillary/inguinal lymph node identification

**FIGURE 1:**
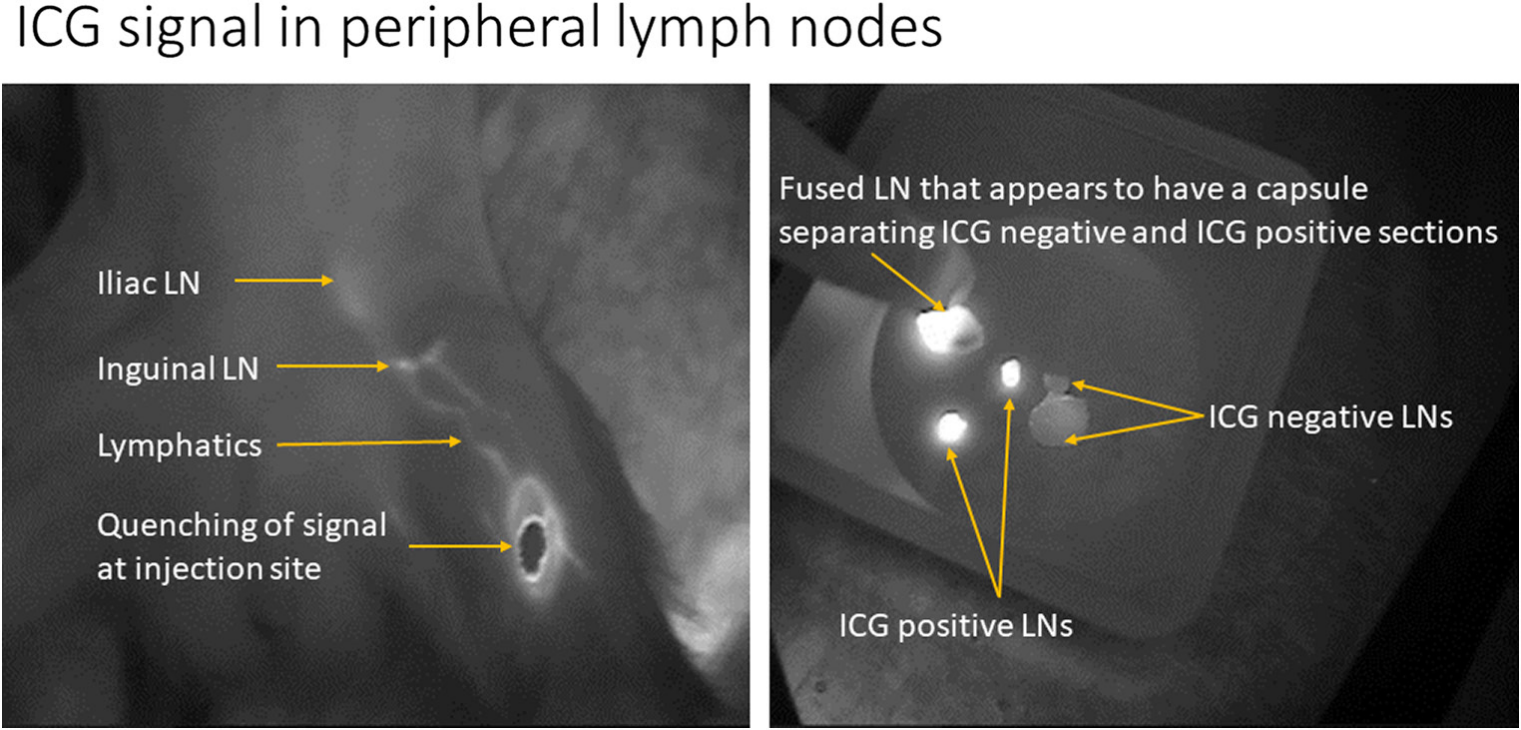
Fluobeam800 image demonstrating the site of ICG injection with quenching. Note the draining lymphatics and ability to detect the inguinal and iliac LNs using DGLN. Collected LNs demonstrate variable fluorescence signal with positive, negative, and a fused node with positive and negative portions present demonstrating the importance of DGLN targeted collection even in terms of which part of the LN to analyze

**FIGURE 2:**
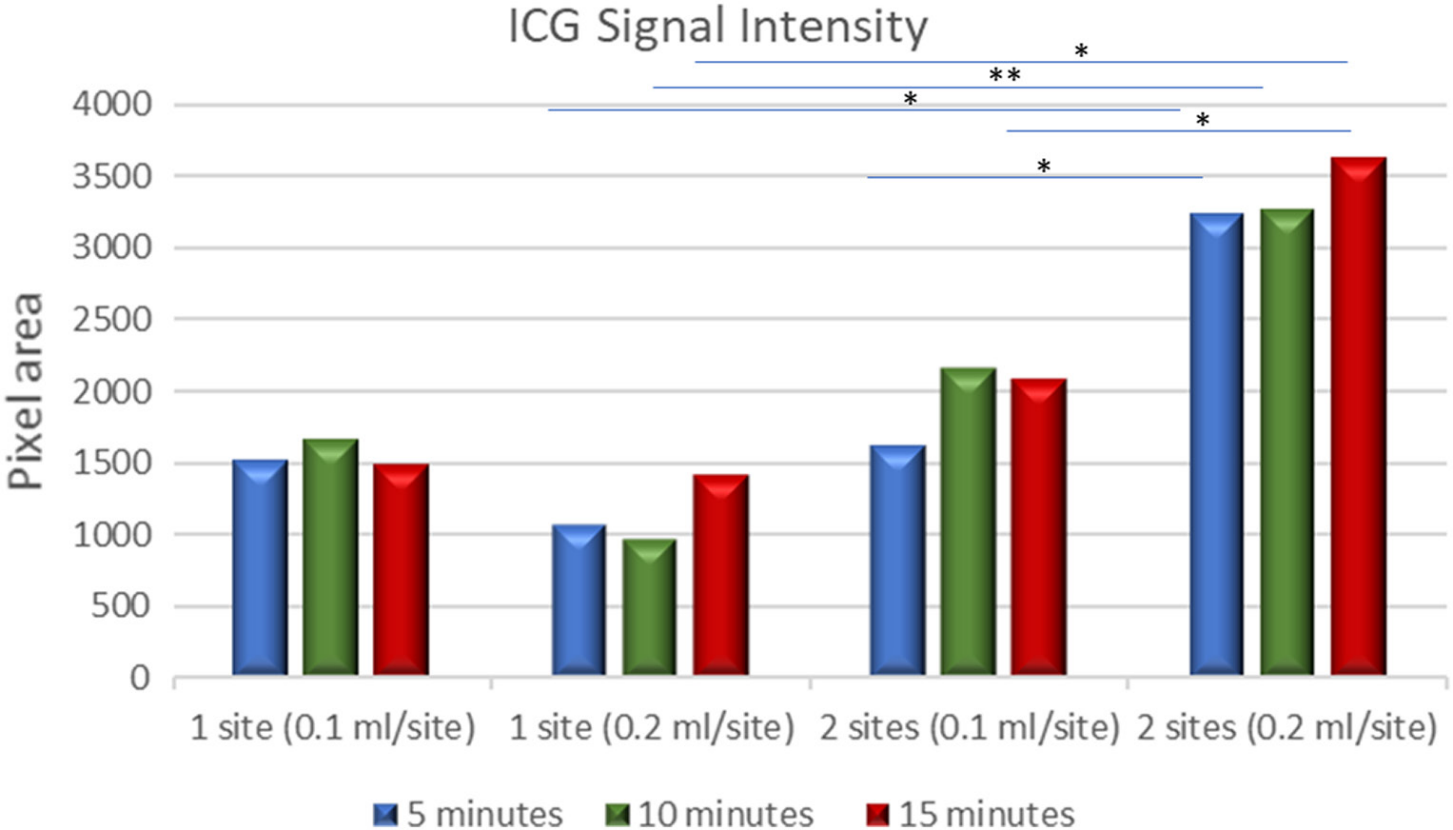
Image J analysis of pixel area of Fluobeam800 images of ICG labeled axillary/inguinal lymph nodes of animals receiving injections at one or two sites with volumes of 0.1–0.2 ml/ site as part of the DGLN optimization process. *p < .05 and **p < .01

ICG was injected at a concentration of 0.625 mg/ml at various locations in the axillary and inguinal areas of macaques as either a single site or at two sites. This concentration was sufficient to allow for identification of multiple LNs down the lymphatic draining chain of nodes and had a mild quenching effect at the center of the injection bleb when imaged (see Figure 1). Together, these findings demonstrated that there was sufficient signal present for identification of LNs and that further increases could actually lessen the signal intensity due to quenching.^14^ For identification of nodes, ICG injection at two sites was superior to one site with a signal intensity of threefold greater at 5 min (*p* = .032), 3.3‐fold greater at 10 min (*p* = .007), and 2.6‐fold greater at 15 min (*p* = .013) when one site on the limb (arm or leg) and one site on the chest/abdomen were used for axillary and inguinal, respectively, at a 0.2 ml volume of ICG (see Figure 2). We determined that a 0.2 ml volume/site presented the optimal volume to achieve signal both in terms of time and signal intensity (*p* = .047 at 5 min and *p* = .018 at 15 min). We then assessed the signal intensity in the nodes at different times post 2 site ID administration of 0.2 ml of ICG and determined that 5 min was optimal through visual assessment.

We then assessed the numbers of LNs at each site with and without fluorescence at necropsy for animals that underwent the 0.2 ml/site x 2 site ICG injections. Animals had between 2 and 14 nodes present per axillary/inguinal site with an average of 77% (50%–100%) of the nodes present at the site demonstrating detectable fluorescent signal using the Fluobeam800, and all animals had at least 2 nodes that were positive at each site. Thus, optimization of our dye‐guided lymph node (DGLN) collection technique resulted in 0.2 ml/site x 2 site ICG injections with imaging performed at least 5 minutes post ID administration for PLN detection.

### 3.2 Use of ICG for PLN and MLN collection in obese macaques

**FIGURE 3:**
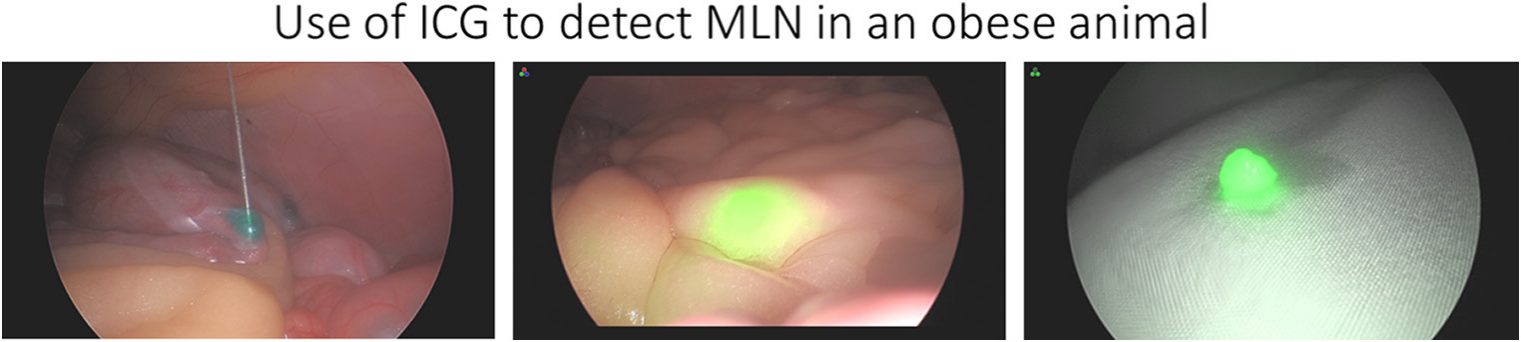
Demonstration of the use of ICG and the Stryker AIM 1688 imaging platform for laparoscopic DGLN identification and collection of MLN in an obese rhesus macaque. From left to right the images represent subserosal injection of 0.4 ml of ICG in white light mode, detection of the fluorescent signal in an MLN draining the site of ICG injection in the mesenteric fat using the overlay mode that captures both white light and the NIR signal, and finally the collected node (also in overlay mode) demonstrating the ability to confirm collection after removal

ICG as optimized for PLN DGLN collection was used in a total of 7 obese rhesus macaques after traditional methods for identification of axillary/inguinal LNs had failed. PLN were identified and collected in 100% (7/7) using DGLN resulting in an average lymphocyte yield of 31.1 × 10^6^/animal (0.8–120 × 10^6^). For MLN, the same concentration of ICG was used for DGLN but was injected at ~0.4 ml/site at 2–3 sites subserosally in the colon (see Figure 3). After ~5–10 min, MLN were visualized using the Stryker1688 and were collected in 100% (9/9) obese rhesus macaques where traditional methods of MLN identification had failed resulting in an average lymphocyte yield of 54.1 × 10^6^/animal (8–185 × 10^6^).

### 3.3 Comparison of draining to nondraining lymph nodes

**FIGURE 4:**
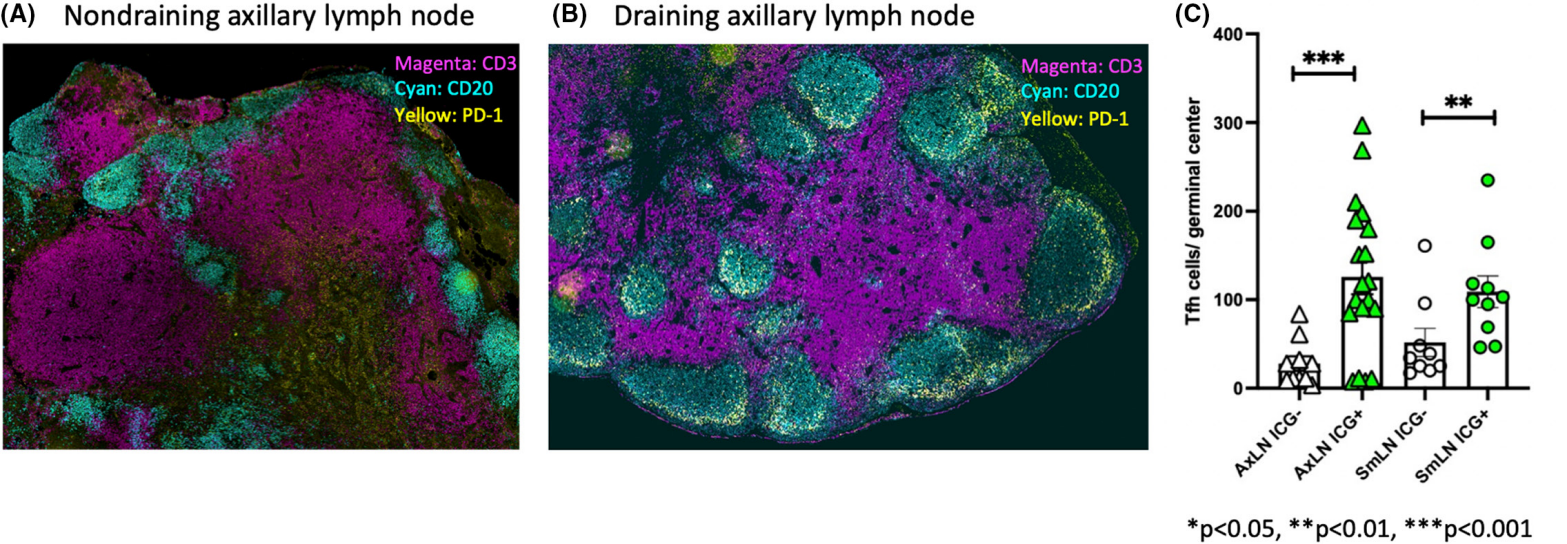
Immunofluorescent antibody staining of draining and nondraining submandibular (SmLN) and axillary (AxLN) lymph nodes 2 weeks after second immunization with HIV Env SOSIP trimer vaccine. Colocalized cells positive for CD3 (magenta) and PD‐1 (yellow) within the germinal centers were identified as T follicular helper (Tfh) cells (CD20+ B cells are also indicated to assist in identification of B cell follicles, Cyan). Representative nondraining, ICG‐ (A) and draining, ICG+ (B) AxLNs are depicted. Tfh cell were quantified from stitched images of biopsy cross sections and average number of Tfh cells per active germinal center were compared in draining and nondraining biopsies (C) following both intraepithelial (SmLN) and intramuscular (AxLN) vaccination

NIR imaging was utilized to target LNs draining the site of an HIV Env SOSIP immunization in two rhesus macaques, one immunized via IM injections and a second via an IEP injections to the oral mucosa. Two doses of vaccine were administered 8 weeks apart. Two weeks after the second vaccination, either axillary (in IM vaccinated) or submandibular (in orally vaccinated) lymph nodes were obtained. NIR DGLN imaging was utilized to identify both nondraining (Figure 4A) and draining (Figure 4B) lymph nodes in each of these areas (designated ICG‐ and ICG+, respectively, Figure 4C). Increased size and number of germinal centers (GCs) was observed in the draining LNs (Figure 4A compared to B). In addition, the number of T follicular helper (Tfh) cells within germinal centers was quantified by immunofluorescent antibody analysis, identifying a significantly higher level of Tfh cells in germinal centers of draining (ICG+) when compared to nondraining LNs within both the axillary (*p* < .001) and submandibular (*p* < .01) locations. The elevated size and number of GCs, as well as increased Tfh cell levels in ICG+ lymph nodes, strongly suggest vaccine‐specific responses are occurring at tissue‐draining lymph nodes and can be identified by the ICG infrared imaging.

## 4 DISCUSSION

We demonstrated the utility of our optimized DGLN technique using ICG, an FDA‐approved fluorescent dye that has both an extremely good safety profile and minimal potential to impact immune responses, for identification of both PLN and MLN in rhesus macaques. This resulted in a 100% rate of identification of LNs that could not be identified by traditional methods. Avoiding missed timepoints is key not only for ensuring that missed samples from unique animals do not skew results, but also to ensure that sufficient numbers are present to achieve statistical significance. Most macaque studies are only powered sufficiently to allow for statistical comparisons when the data set is complete, and missing samples that require elimination of an animal from a timepoint or from the study can result in a loss of the statistical power required to adequately answer an experimental question. We have demonstrated that these fluorescence DGLN techniques can be combined with other techniques to permit a more comprehensive evaluation of changes in the immune system over time in macaque models. It is not uncommon for us to perform comprehensive evaluations inclusive of PLN, MLN, spleen, liver, bone marrow, bronchoalveolar lavage, and mucosal biopsies (duodenum, colon, vagina, and/or oral mucosa) collected during the same anesthetic event at up to 8 timepoints during the course of a study. This is performed using minimally invasive techniques that optimize animal welfare, minimize impacts to the animals and associated experimental results, and allow for longitudinal comparisons within an animal avoiding the inter‐animal variability seen in animal intense serial sacrifice studies.

Additionally, we demonstrated the ability to use ICG DGLN to identify draining versus nondraining LNs present at axillary and submandibular sites. These initial analyses utilizing one macaque vaccinated via the oral IEp route, and one vaccinated via the IM route were able to provide evidence that ICG‐positive LNs draining the site of immunization were distinct from those that were physically adjacent but ICG‐negative. Indeed, both greater numbers/size of B cell follicles were observed in the ICG‐positive LNs, as well as higher levels of Tfh positive cells in the germinal centers of these follicles. The levels of Tfh likely represent the proliferation of these cells following exposure to the SOSIP trimer vaccine, Tfh cells then stimulate B cells to expand and eventually produce antibodies. Importantly, the identification of nodes with extensive germinal center formation provides confidence that we were able to correctly identify the active draining lymph nodes responding to vaccination, allowing us to selectively harvest the sites of immunologic importance for downstream analyses.

These DGLN techniques can be employed either using a labeled target (antibody, antigen, virus, etc.)^15^, ^16^, ^17^ or by injecting unbound ICG at the same site^18^, ^19^ or both using the signal from the labeled target to guide administration of the unbound ICG which in turn serves to increase the signal intensity in the draining LN. Identification of actual draining LNs is key for any localized response,^20^, ^21^, ^22^, ^23^ such as an immunization, local infection, or early local viral spread after a mucosal exposure. Early events in macaque models of SIV have been difficult to assess due to inter‐animal variability and/or the need for serial sacrifice at early timepoints that do not permit following an animal to determine disease progression and kinetics.^10^, ^24^, ^25^ Serial comprehensive sampling, especially using targeted DGLN collections, could permit evaluation of differences in responses that lead to different viral kinetics or differences in progression to systemic infection vs local control and eventual elimination overtime. Following responses, overtime from preinfection to setpoint viremia will be essential for optimizing responses to vaccines and therapeutics aimed at early intervention to reduce reservoirs and setpoint viremia in SIV models designed to inform clinical trials in HIV patients. In addition to providing better data, avoiding unnecessary animal intense serial sacrifice studies will also result in a reduction in the number of animals required to address an experimental question. Given that the MIS techniques have very low complication rates (typically <1%)^4^ and do not require the use of anti‐inflammatory or antibiotic medications the main limitations to their use are equipment and surgeon training.

The use of NIR imaging is ideal for DGLN identification given the long wave length which leads to greater tissue penetration when compared to other fluorescence imaging techniques. ICG is ideally suited as a DGLN contrast agent for use in NIR detection of draining LN given the long (>800 nm) wavelength of emission and the corresponding depth (~1 cm) of tissue fluorescence signal penetration.^26^, ^27^, ^28^ We observe minimal to no autofluorescence in this range, allowing for excellent signal‐to‐noise ratios. Because of the lipoprotein binding capacity of ICG and the high protein content of lymph, ICG accumulates in the lymphatic pathways and LNs.^29^ Additionally, given its small size (~775 daltons) ICG is extremely unlikely to generate immune responses. These characteristics also make ICG superior to other techniques involving nonfluorescent inks/dyes that can be mistaken for natural pigments, such as hemosiderin, blood accumulation in the LN, or the presence of tattoo ink from animal identification, and which have been shown to have inflammatory properties.^30^, ^31^, ^32^ The movement of ICG from the site of injection to the primary draining LN occurs very quickly, with 5 minutes representing the ideal balance between wait time and signal intensity based on the optimization work we have done. Additionally, if the ICG is administered early in the process of preparing the site for surgery, there is no additional time required for the procedure as it takes ~5 minutes to shave, prep, and drape the site in most cases.

In conclusion, we have developed NIR DGLN techniques for identification of PLN and MLN, providing a powerful tool that can be used to study immunity in macaques with a high degree of accuracy and specificity. Applications that analyze lymph node‐specific responses are heavily dependent on accurate recovery of immunologically active lymphoid tissue and thus will greatly benefit from the incorporation of NIR‐guided LN identification. More broadly, because ICG can be coupled to many types of molecules, this technique is compatible with virtually any approach aimed at identifying trafficking of biologics or compounds to the lymph nodes, creating the opportunity to selectively and accurately analyze outcomes of LN‐specific interventions.

## CONFLICT OF INTEREST

The authors do not have any conflicts of interest in regards to this publication.

## ETHICS STATEMENT

This study was performed in accordance with the ethical policies of the journal, and in compliance with Guide for the Care and Use of Laboratory Animals, the USDA Animal Welfare Regulations and the Public Health Service Policy on Humane Care and Use of Laboratory Animals.

## Data Availability

The data that support the findings of this study are available from the corresponding author upon reasonable request.
